# Int6/eIF3e Is Essential for Proliferation and Survival of Human Glioblastoma Cells

**DOI:** 10.3390/ijms15022172

**Published:** 2014-01-29

**Authors:** Julie Sesen, Anne Cammas, Sarah J. Scotland, Bertand Elefterion, Anthony Lemarié, Stefania Millevoi, Lijoy K. Mathew, Cathy Seva, Christine Toulas, Elizabeth Cohen-Jonathan Moyal, Nicolas Skuli

**Affiliations:** 1 INSERM U1037, Centre de Recherche en Cancérologie de Toulouse, 20-24 Rue du Pont St Pierre, 31052 Toulouse, Cedex, France; E-Mails: julie.sesen@hotmail.fr (J.S.); anne.cammas@inserm.fr (A.C.); sarah.scotland1@gmail.com (S.J.S.); elefterion.bertrand@live.fr (B.E.); lemarie.anthony@claudiusregaud.fr (A.L.); stefania.millevoi@inserm.fr (S.M.); cathy.seva@inserm.fr (C.S.); toulas.christine@claudiusregaud.fr (C.T.); moyal.elizabeth@claudiusregaud.fr (E.C.-J.M.); 2 Abramson Family Cancer Research Institute, University of Pennsylvania, Philadelphia, PA 19104, USA; E-Mail: mathewl@mail.med.upenn.edu; 3 Département de Radiothérapie Institut Claudius Regaud, 20-24 Rue du Pont St Pierre, 31052 Toulouse, Cedex, France

**Keywords:** glioma, glioblastoma, proliferation, apoptosis, eukaryotic translation initiation factor, Int6, eIF3e, Hypoxia Inducible Factors, HIF-1α, HIF-2α

## Abstract

Glioblastomas (GBM) are very aggressive and malignant brain tumors, with frequent relapses despite an appropriate treatment combining surgery, chemotherapy and radiotherapy. In GBM, hypoxia is a characteristic feature and activation of Hypoxia Inducible Factors (HIF-1α and HIF-2α) has been associated with resistance to anti-cancer therapeutics. Int6, also named eIF3e, is the “e” subunit of the translation initiation factor eIF3, and was identified as novel regulator of HIF-2α. Eukaryotic initiation factors (eIFs) are key factors regulating total protein synthesis, which controls cell growth, size and proliferation. The functional significance of Int6 and the effect of *Int6/EIF3E* gene silencing on human brain GBM has not yet been described and its role on the HIFs is unknown in glioma cells. In the present study, we show that Int6/eIF3e suppression affects cell proliferation, cell cycle and apoptosis of various GBM cells. We highlight that Int6 inhibition induces a diminution of proliferation through cell cycle arrest and increased apoptosis. Surprisingly, these phenotypes are independent of global cell translation inhibition and are accompanied by decreased HIF expression when Int6 is silenced. In conclusion, we demonstrate here that Int6/eIF3e is essential for proliferation and survival of GBM cells, presumably through modulation of the HIFs.

## 1. Introduction

Based on the World Health Organization (WHO) classification, Glioblastoma Multiforme (GBM) are grade IV astrocytic brain tumors [1]. They are the most common brain tumors in adults, representing the second cause of death in children and the third in adults. GBM are one of the most deadly human cancers and despite surgery, radiotherapy and chemotherapy, the median survival is approximately 12–14 months. Furthermore, these aggressive tumors are known to be hypoxic which could contribute for their resistance to anti-cancer therapeutics [2,3].

Indeed, cell response and adaptation to low oxygen conditions is primarily controlled by Hypoxia Inducible Factors (HIFs). These transcription factors act as a heterodimer, composed of two subunits: HIF-α and HIF-β. The two major HIF-α subunits are HIF-1α and HIF-2α and these factors mediate cellular adaptation to hypoxia [4]. The main regulation of the α subunits is the oxygen tension however their stabilization and consequent functions are also influenced by genetic alterations, cell metabolism, growth factors, cytokines and hormones [5]. HIFs promote adaptation to hypoxic conditions through the regulation of more than 150 genes involved mainly in angiogenesis, metabolism, proliferation and cell migration [4,6]. HIF-2α plays a particular role in GBM cells and glioma stem cells and is highly expressed in these cells [7,8]. HIF-2α can be active under non-hypoxic conditions creating a pseudo-hypoxic phenotype that influences cell behaviors. Its knockdown has been show to prevent glioma cell growth *in vitro*, which is associated with reduced levels of VEGF (Vascular Endothelial Growth Factor) as well as, poorly vascularized and highly necrotic tumors *in vivo* [7].

Recently, Int6, also known as eIF3e (“e” subunit of the eukaryotic translation Initiation Factor 3), has been described as a new regulator of HIF-2α [9–12]. Int6/eIF3e, through the Eukaryotic Initiation Factor 3 (eIF3), is mainly involved in protein synthesis, due to its direct binding to the 40S ribosome and facilitating ribosome recruitment to mRNA [13,14]. The core of eIF3 is composed of eIF3a, eIF3b, eIF3c, eIF3g and eIF3i, while eIF3e, eIF3f and eIF3h have been shown to stabilize the main core and modulate its activity [15,16]. Interestingly, it has been shown that some of these eIF3 subunits play a role in tumorigenesis [13,14]. Despite altered expression in different cancer types, eIF3e’s involvement in tumorigenesis is not yet clear. Of note, Int6 has other surprising functions such as contributing to the DNA damage response in HeLa cells through involvement of ATM and BRCA1 [17]. In breast carcinoma cells, Int6 depletion induces diminished proliferation, decreasing urokinase-type plasminogen activator (PLAU) and apoptotic regulator BCL-XL [18], and favors epithelial-to-mesenchymal transition increasing Snail and Zeb2 expression [19]. Finally, Int6 modulates HIF-2α expression and its target genes to control vascular remodeling and development [11,12].

To date, Int6/eIF3e expression in human glioma cells and its role in cell growth have not been studied. The aim of the present work was to determine the *in vitro* effect of *Int6/EIF3E* gene silencing by RNA interference on a panel of human GBM cell apoptosis and cell cycle and to elucidate its molecular mechanism potentially through HIF modulation.

## 2. Results and Discussion

### 2.1. Results

#### 2.1.1. Int6/eIF3e Expression in Human Glioblastoma Cells

**Figure 1.**
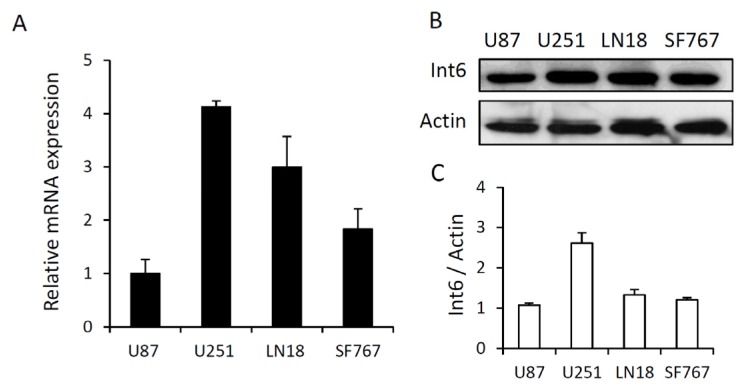
Basal Int6/eIF3e expression in four different glioblastoma cell lines. (**A**) Graph representing *Int6* mRNA levels in LN18, SF767, U87 and U251 glioma cells analyzed by qRT-PCR (*n* = 4); (**B**) Western blot analysis showing basal Int6 protein expression in LN18, SF767, U87 and U251 glioma cells (*n* = 5); (**C**) Western blot quantifications showing the ratio Int6/eIF3e/Actin of at least 5 independent experiments.

First, we analyzed Int6 expression in four different GBM cell lines (LN18, SF767, U87 and U251) by qRT-PCR and western blot analysis. qRT-PCR analyses revealed that *Int6* mRNA is highly expressed in all glioma cell lines tested. U251 cells exhibit the highest mRNA expression and U87 cells the lowest (Figure 1A). In addition, basal Int6 protein expression was assessed by western blot and is partly correlated with *Int6* mRNA expression. The U251 cells have the strongest Int6/eIF3e expression while the U87 cells have the lowest within the four different glioma cell lines (Figure 1B,C). These results show that Int6/eIF3e is well expressed in GBM cells and some differences between cell lines are observed.

#### 2.1.2. RNA Interference Mediated *Int6/EIF3E* Silencing in Glioblastoma Cells

**Figure 2.**
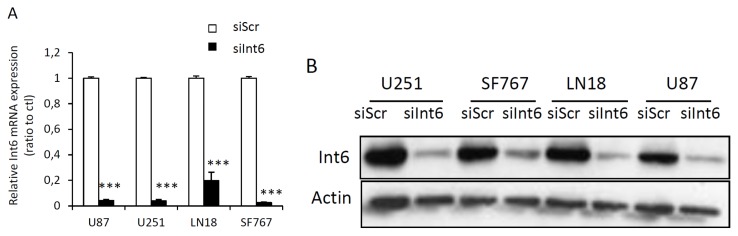
Efficient inhibition of Int6/eIF3e expression with siRNA. (**A**) qRT-PCR data showing strong *Int6* mRNA depletion in GBM cells transfected with *Int6* specific siRNA (siInt6) compared to control cells transfected with a scrambled sequence (siScr) (*** p < 0.001 siInt6 *versus* siScr, *n >* 4); (**B**) Western blots showing efficient inhibition of Int6/eIF3e protein expression in LN18, SF767, U87 and U251 GBM cells transfected with *Int6* specific siRNA (siInt6) (*n =* 10).

Using an RNA interference strategy, we tested different concentrations of control siRNA (siScr) or specific siRNA for *Int6/EIF3E* (siInt6) and performed a time course experiment in order to determine the efficiency of the siRNA over time. We show that siInt6 strongly and specifically inhibits *Int6* mRNA and protein in all GBM cell lines compared to control siRNA (Figure 2 and Figure S1). The range of 1 nM to 50 nM of specific siRNA gave us a complete Int6 inhibition and 20 nM continued to inhibit Int6 seven days post transfection (Figure S1A,B). Of note, in order to be confident that the phenotypic results we observed were due to knockdown of the target of interest and not due to an off-target effect, we repeated some of the experiments described below with different and distinct siRNAs, targeting *Int6/EIF3E*, in parallel to confirm our results (see experimental section and Figure S1C–E and Figure S2A,B). These results demonstrate that siRNA transfection silenced *Int6* gene and protein expression effectively.

#### 2.1.3. Int6 Inhibition Decreases Glioblastoma Cell Proliferation

**Figure 3.**
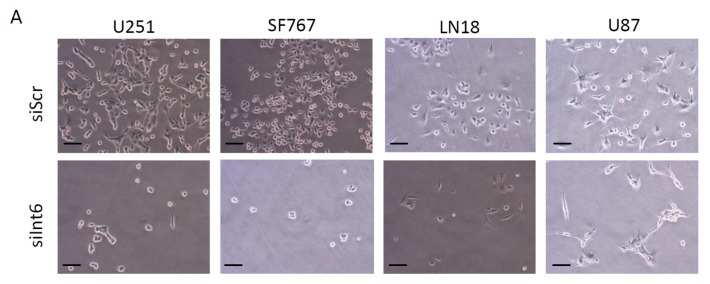

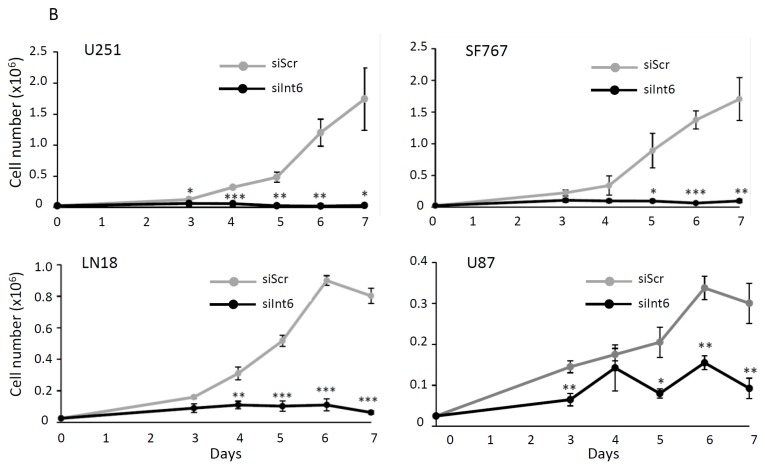
Int6/eIF3e inhibition suppresses GBM cell proliferation. (**A**) Photographs taken during the proliferation assay and representing LN18, SF767, U87 and U251 GBM cells 72 h after transfection with siInt6 or siScr (scale bars: 20 μm); (**B**) Proliferation assay performed with each GBM cell lines (U251, SF767, LN18 and U87) showing a significant decrease in cell number when cells are transfected with siInt6 (grey curve: control siScr, black curve: siInt6) (* p < 0.05, ** p < 0.01, *** p < 0.001 siInt6 *versus* siScr, *n >* 4).

To investigate whether RNA interference-mediated *Int6/EIF3E* gene silencing affects glioblastoma cell growth and proliferation, we transfected LN18, SF767, U87 and U251 cells with control siRNA (siScr) or specific siRNA for *Int6* (siInt6) and followed cell growth by counting cells daily for 7 days. Three days after transfection, we observed a significant and strong decrease in cell number when *Int6* is silenced (siInt6) compared to controls in all GBM cells tested (Figure 3A,B and Figure S2). These results indicate that Int6 is essential for cell growth and proliferation of GBM cells.

#### 2.1.4. Int6 Inhibition Induces Cell Cycle Arrest and G0 Transition

**Figure 4.**
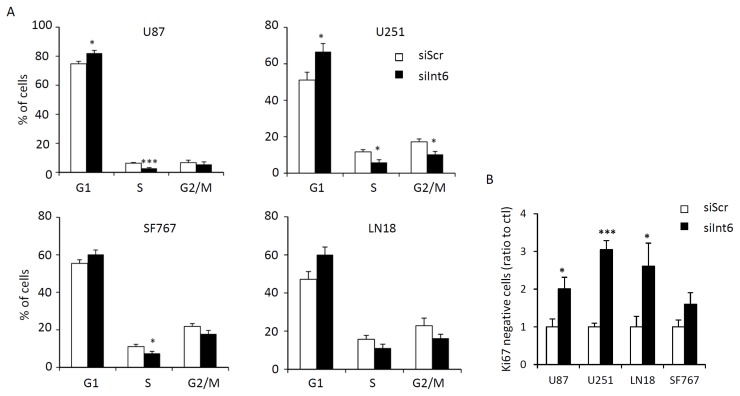
Int6/eIF3e inhibition halts GBM cell cycle. (**A**) Quantification of cell cycle distribution of GBM cells using flow cytometry and PI staining. *Int6* knockdown (siInt6) induces decreased cell number in S phase and increased cell number in G1 phase compared to control cells (siScr); (**B**) Quantification of GBM cells in G0 phase using flow cytometry and Ki67/PI staining. *Int6* knockdown significantly increases the number of cells in G0 phase (* *p* < 0.05, *** *p <* 0.001 compared with the negative control, siScr, *n =* 4).

In order to explain the decreased proliferation with Int6 inhibition, we first looked at cell cycle and cell cycle arrest in human GBM cells. Using PI/Ki67 staining and flow cytometry, we analyzed cell cycle and cells in G0. We demonstrate that Int6 inhibition leads to a modest but significant block in the G1 phase (Figure 4A and Figure S3A). Cells transfected with siInt6 accumulated more in G1 (siInt6: 82.10% for U87, 66.66% for U251, 60.22% for SF767 and 60.10 for LN18) compared to cells transfected with control siRNA (siScr: 74.74% for U87, 55.00% for U251, 54.41% for SF767 and 47.18% for LN18). In correlation with this G1 block, we also observed an increase in the number of cells in G0 when Int6 was inhibited. The percentage of Ki67 negative cells was at least twice higher in response to *Int6* silencing (Figure 4B and Figure S3B). These results indicate that inhibition of GBM cell growth by *Int6* gene silencing may induce G1 phase cell cycle arrest and transition in G0.

#### 2.1.5. Int6 Inhibition Increases Glioblastoma Cell Apoptosis

**Figure 5.**
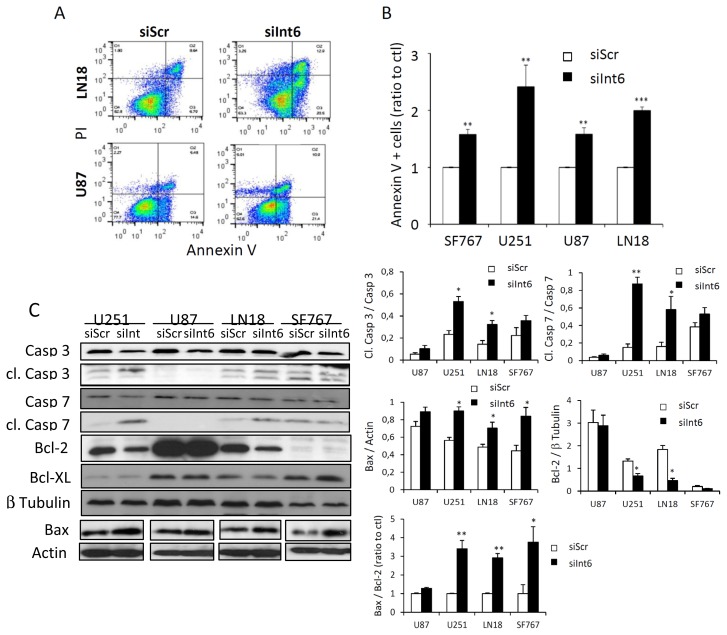

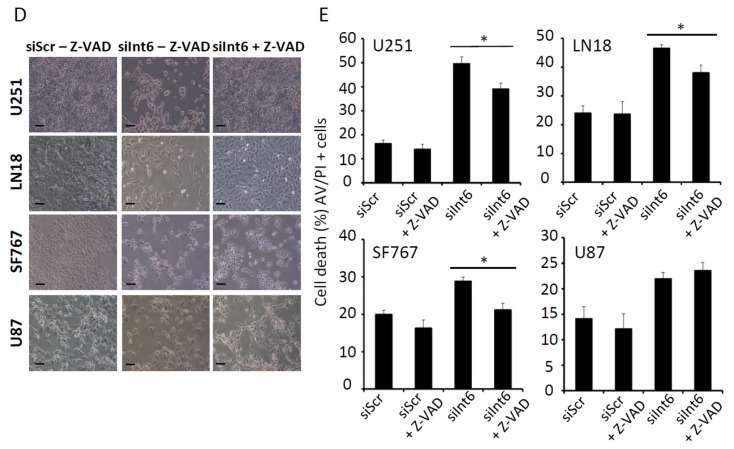
*Int6* gene silencing induces cell apoptosis. (**A**) Dot plot representing the percentage of Annexin V positive cells in two GBM cell lines (LN18 and U87) analyzed by flow cytometry and Annexin V/Propidium Iodide staining; (**B**) Quantification of Annexin V positive cells. Int6 inhibition (siInt6) results in a significant increase in apoptotic cells (** *p* < 0.01, *** *p <* 0.001 compared with the negative control, siScr, *n =* 5); (**C**) Western Blot analysis and quantifications of caspase 3, cleaved caspase 3, caspase 7, cleaved caspase 7, Bcl-2, Bcl-XL and Bax expression in LN18, SF767, U87 and U251 GBM cells transfected with siInt6 or siScr. *Int6* silencing slightly increases Bax expression, reduces Bcl-2, caspase 3 and caspase 7 expression, and increases Bax/Bcl-2 ratio and cleaved caspase forms for U251 and LN18 cells (* *p* < 0.05, ** *p* < 0.01, *n* = 3); (**D**) Photographs representing glioma cells transfected with siScr or siInt6 and treated with 30 μM of Z-VAD caspase inhibitor (+Z-VAD) or DMSO (−Z-VAD). Z-VAD treatment partly rescues the deleterious effect of Int6 silencing (*n* = 3), scale bars: 20 μm; (**E**) Quantification of Annexin-V/PI staining analyzed by flow cytometry of glioma cells transfected with siScr or siInt6 and treated or not with Z-VAD caspase inhibitor. Z-VAD treatment partly reverses glioma cell death induced by Int6 inhibition (* *p* < 0.05, *n* = 3).

Apoptosis often is a consequence of the G0/G1 arrest and could be another explanation for the effect of Int6 inhibition on glioma cell proliferation. We decided to assess apoptosis rate by flow cytometry and western blot in the four different GBM cell lines using Annexin-V/PI staining and apoptosis-related proteins such as caspase 3, caspase 7, PARP, Bcl-XL and Bax, respectively. Three days following RNA interference-mediated *Int6* gene silencing (siInt6), the apoptosis rate, reflected by the percentage of AnnexinV positive cells, was significantly increased compared to control cells (siScr) (Figure 5A,B and Figure S4A). In addition, we confirmed the apoptotic process by studying caspase 3, caspase 7, PARP, Bcl-XL and Bax expression in our cell lines. All cell lines exhibit a slight decrease in total caspase 3, caspase 7 and PARP expression and a slight increase in expression of the pro-apoptotic protein Bax (Figure 5C and Figure S4B,C). Surprisingly, only U251 and LN18 cells show a significant increase in the cleaved caspase forms, suggesting a specific and prominent role of caspases in the apoptotic phenotype of these cell lines (Figure 5C). Additionally, these cell lines also exhibit a significant decrease of the anti-apoptotic protein Bcl-2 (Figure 5C). The Bax/Bcl-2 ratio was significantly increased in U251, LN18 and SF767 revealing an apoptotic mechanism (Figure 5C), and only a trend was observed for the U87 cell line. Since previous work has identified Bcl-XL to be regulated by eIF3e, we also assessed Bcl-XL expression in response to *Int6/EIF3E* silencing [18]. Interestingly, we did not observe any difference in Bcl-XL expression in our glioma cells when Int6/eIF3e was depleted (Figure 5C). To further understand the caspase involvement in our phenotypes, we assessed caspase-dependent cell death using the Z-VAD caspase inhibitor in glioma cells transfected or not with siInt6 (Figure 5D,E). Flow cytometry analyses of Annexin-V/PI staining demonstrate that Z-VAD treatment induced a partial, but significant reversal of glioma cell death for U251, LN18 and SF767 cells confirming a caspase role in glioma cell apoptosis when Int6 is inhibited (Figure 5D,E). Based on cleaved caspase expression and Z-VAD experiments, the U87 cell line seems to induce a caspase-independent apoptotic mechanism. These observations indicate that Int6 inhibition in human glioma cells induces apoptosis through caspase-dependent and caspase-independent pathways.

#### 2.1.6. RNA Interference-Mediated *Int6/EIF3E* Silencing, Translation and HIFs

**Figure 6.**
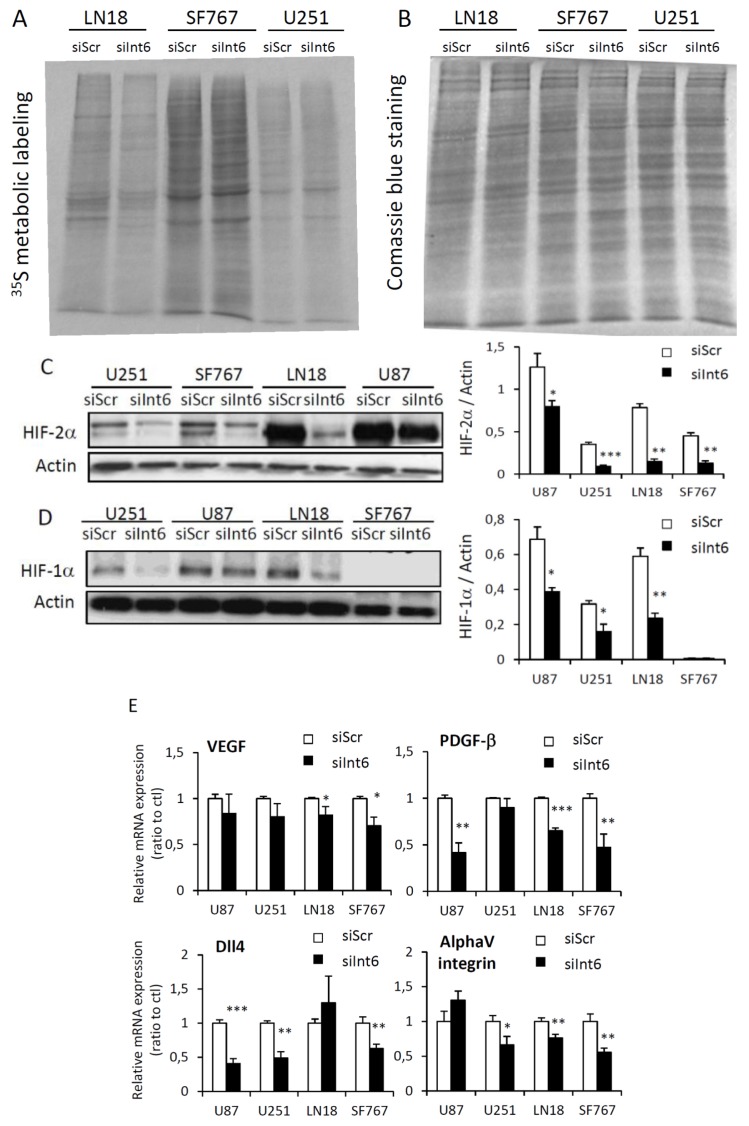
*Int6* gene silencing downregulates the Hypoxia Inducible Factors expression without affecting global cell translation. (**A**,**B**) *De novo* protein synthesis in GBM cell lines (72 h after siScr or siInt6 transfection) was assessed by ^35^S Methionine metabolic labeling. Proteins were resolved by SDS-polyacrylamide gel, autoradiographed (**A**) and stained with Coomassie (**B**); (**C**,**D**) Western blot analysis of HIF-2α (**C**) and HIF-1α (**D**) expression in human GBM cells transfected or not with siInt6 showing decreased HIF expression when *Int6/EIF3E* is knockdowned, *n =* 5; (**E**) Graphs representing HIF target gene (*VEGF*, *PDGF-β*, *Dll4*, *αV integrin*) expression in control or *Int6* silenced GBM cells, assessed by qRT-PCR. Int6 inhibition significantly reduces HIF target gene expression. (* *p* < 0.05, ** *p <* 0.01, *** *p <* 0.001, *n =* 4).

Int6/eIF3e is a component of the eukaryotic initiation factor 3 (eIF3), which is required for several steps in the initiation of protein synthesis. *Int6* silencing in our glioma cell lines led to a strong inhibition of proliferation. We analyzed whether Int6/eIF3e inhibition was affecting global cell translation. Using ^35^S-Methionine metabolic labeling, we assessed *de novo* protein synthesis in LN18, SF767 and U251 cells (Figure 6A,B and Figure S5B,C). Interestingly, we did not observe any major difference in translation profiles between control cells (siScr) and cells where *Int6* was silenced (siInt6) (Figure 6A,B). Furthermore, we did not observe any difference in protein expression profile on a coomassie blue stained gel loaded with proteins coming from the same number of control or cells transfected with siInt6 (Figure S5A). These results suggest that Int6/eIF3e depletion does not affect global protein synthesis in human GBM cells. Int6/eIF3e has also been identified as a potential regulator of the Hypoxia Inducible Factors, particularly HIF-α [6]. Based on the role of the HIFs in promoting metabolism, migration, proliferation and cell growth in glioma, we decided to assess HIF status and their target genes in our human GBM cell lines by western blot and qRT-PCR. *Int6* silencing has been shown to promote HIF-2α stability, however and very surprisingly, in human glioma cells, Int6 inhibition leads to a significant decrease of HIF-1α and HIF-2α expression with a more pronounced down-regulation of HIF-2α (Figure 6C,D and Figure S1), particularly in U251, LN18 and SF767 cells. We also assessed several HIF target genes by qRT-PCR. HIF reduced expression was correlated with diminished *Vascular Endothelial Growth Factor* (*VEGF*), *Platelet-Derived Growth Factor β* (*PDGF-β*), *AlphaV integrin* (*αV integrin*) and *Delta Like Ligand 4* (*Dll4*) mRNAs (Figure 6E). Specifically, *VEGF* mRNA level is slightly decreased in all glioma cell lines when *Int6* is silenced. With siInt6, *PDGF-β* mRNA is down-regulated in LN18, U87, and U251 cells, *αV integrin* mRNA is decreased in U251, LN18, and SF767 cells, and *Dll4* mRNA is down-regulated in SF767, U87, and U251 cells. These results demonstrate that *Int6* silencing, and consequently Int6/eIF3e inhibition, decreased HIF expression and their transcriptional activity and that Int6/eIF3e could be a potential positive HIF regulator in human GBM cells.

### 2.2. Discussion

Glioblastoma (GBM) are very hypoxic, aggressive and resistant tumors and identification of new biological pathways is crucial to improve anti-cancer therapeutics [1,2]. Hypoxia Inducible Factors (HIFs), particularly HIF-2α, are important regulators involved in GBM resistance through the control of cell response and adaptation to low oxygen conditions. Targeting the HIFs remains challenging and several teams are currently involved in the development of specific inhibitors of HIF-1α and HIF-2α [5]. Recent research on translation and protein synthesis in malignant tumors also demonstrates the key role played by translational control in tumorigenic processes [13]. Our study identified a new biological pathway regulated by Int6/eIF3e, which modulates GBM cell growth and proliferation. Int6/eIF3e was recently identified as a novel regulator of HIF-2α [6,9] and is primarily known to form, with 12 other subunits, the Eukaryotic Initiation Factor 3 (eIF3) which facilitates the interaction between ribosome and mRNA [14,16]. The eIF3 consists of five subunits (eIF3a, eIF3b, eIF3c, eIF3g and eIF3i), which represent the essential core for the initiation of translation [15,16]. The remaining subunits have been described as modulators of its activity. Specifically, eIF3e, eIF3f and eIF3h are involved in the stabilization of the main core [16]. Interestingly, it has been shown that eIF3 subunits play a role in tumorigenesis. For instance, eIF3a, eIF3c or eIF3h are overexpressed in many tumors, leading to increased protein synthesis and supporting tumor development, while their inhibition can reverse the malignant phenotypes [20–22]. Recently, Liang *et al.* demonstrated that eIF3b was strongly expressed in human GBM and human GBM cell lines (U251, U373, U87 and A172) [23]. In this study, eIF3b depletion induces glioma cell apoptosis and cell cycle inhibition leading to decreased GBM cell proliferation, which is similar to what we observed with *EIF3E* silencing [23].

The *EIF3E/Int6* gene was originally identified as the site of frequent insertion of the mouse mammary tumor virus [24,25]. However, its role in carcinogenesis is still controversial in the literature and could depend on the tumor stage and type. On one hand, Int6/eIF3e has been described as a potential tumor suppressor with low expression associated with a loss of heterozygosity at the *Int6* locus in breast and lung cancer [26,27]. Supporting these data, eIF3e inhibition, *in vitro*, in human mammary epithelial cells favored transformation [28] and induced epithelial-to-mesenchymal transition [19]. On the other hand, previous studies have reported that *Int6* silencing inhibits the proliferation of various tumor cells suggesting a role for Int6 in tumor progression and development. Particularly, eIF3e depletion in ovarian (HeLa) and breast (MDA-MB-231) cancer cells or osteosarcoma cells (U2OS) induces apoptosis, and consequently leads to an inhibition of proliferation. Int6/eIF3e function in the complex mechanism underlying tumor formation and maintenance is still under debate and nothing is currently known in regards to the effect of Int6/eIF3e inhibition in human glioma cells.

**Figure 7.**
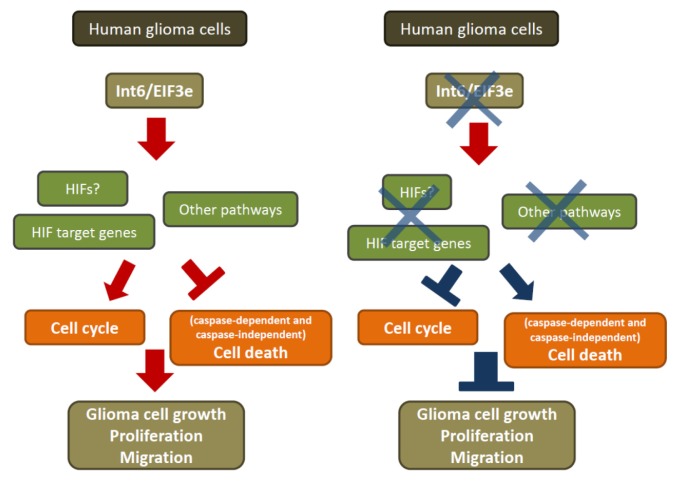
Int6/eIF3e is essential for human glioblastoma cell growth. Int6 inhibition leads to decreased glioma cell proliferation and migration, presumably through decreased HIF expression and activity and increased cell cycle arrest and apoptosis.

Therefore, the present study aimed to determine the role of Int6/eIF3e in glioma cells (Figure 7) and its molecular mechanism. RT-PCR and western blot analysis were performed on four different glioma cell lines (LN18, SF767, U87 and U251) revealing strong *Int6* gene and protein expression. To establish the effect of Int6 on these GBM cells, we used small RNA interfering technology to silence *Int6* expression. Previous studies have shown that *Int6* silencing does not have any impact on MCF-10A cell proliferation [28]. However, Int6 inhibition in HeLa, U2OS and MDA-MB-231 cells results in decreased cell growth [17–19,28,29]. Following Int6 inhibition in human glioma cells, we observed that proliferation was markedly reduced, indicating that Int6/eIF3e is involved in the regulation of glioma cell proliferation.

Flow cytometry analyses revealed that Int6 depletion arrested glioma cells in the G0/G1 phase. This observation indicates that Int6/eIF3e inhibition leads to a reduction in the number of glioma cells undergoing cell division and increases cell cycle arrest. We also demonstrated that apoptosis occurred in human GBM cells following *Int6/EIF3E* silencing confirming previous studies reporting that Int6 inhibition leads to apoptosis in HeLa cells [17,29]. Consistent with these results, we noticed that caspase pathways were activated, particularly in U251 and LN18 glioma cells, following Int6 inhibition. This activation of members of the caspase family is an important prerequisite for apoptosis and could partly explain our apoptotic phenotype. Furthermore, experiments with Z-VAD caspase inhibitor allowed us to confirm the partial caspase involvement in glioma cell death when *Int6/EIF3E* is silenced. However, for the SF767 and U87 cell lines, the apoptotic process seems to be caspase-independent. The different responses of glioma cells to Int6 inhibition requires clarifications but could potentially be explained by the diverse genetic/metabolic backgrounds of the cells, which were selected for their different mutations found in glioblastomas. Based on these various backgrounds, we did not expect all the cells to behave in the same manner following *Int6* depletion as different cell death pathways could be involved. Our study focused primarily on typical cancer cell phenotypes, such as proliferation, cell cycle and apoptosis. However, glioblastoma cells are also known to be highly invasive. Thus, we also performed migration assays with glioma cells transfected with control or *Int6/EIF3E* specific siRNAs (Figure S6). Although Int6 inhibition in breast epithelial cells (MCF-10A) has been demonstrated to induce a constitutive migratory phenotype suggesting a role for Int6 in epithelial-mesenchymal transition [19], we observed that *Int6/EIF3E* silencing led to a decreased migration in our glioma cell lines. These results are currently under further investigation to explain this discrepancy.

It has been shown that Int6 regulates the MAPK pathway through control of MEK expression during zebrafish development [30]. This mechanism could explain the proliferation defect we observed following a knockdown of *Int6*. However, we did not observe any significant differences in activation of the MAPK pathway in our glioma cells, demonstrating that *Int6* silencing inhibits cell proliferation independently of the MAPK pathway (Figure S7). Based on the Int6/eIF3e role within eIF3 and translation, we also assessed translation and protein synthesis in human glioma cells [13]. Impairment of protein synthesis could explain the strong cell growth inhibition. Very interestingly, we did not notice any difference in translation between control cells and cells where Int6 was inhibited. However, Int6/eIF3e depletion could affect specific mRNA translation without impacting global protein translation. Indeed, our results support the work from Grzmil *et al.* who reported that Int6 inhibition failed to modify the global translation in breast cancer and osteosarcoma cells [18]. In MDA-MB-231 cells, the analysis of polysome-bound mRNA revealed that Int6 positively and negatively regulates specific mRNAs, including *PLAU*, *BCL2L1* (encoding BCL-XL) and *MAD2L1* (MAD2 mitotic arrest deficient-like 1), respectively. Isolation of polysome-bound mRNA from control and siInt6 glioma cells would allow us to identify such mRNAs.

Hypoxia Inducible Factors are essential transcription factor for cellular response and adaption to low oxygen conditions [4]. Inhibition of these factors has been shown to prevent growth, proliferation, migration of human GBM cells *in vitro* and *in vivo* [31–34]. Recently, it has been shown in MCF-7 and endothelial cells that Int6/eIF3e, and particularly its overexpression, induces decreased HIF-2α expression. In contrast, its silencing promotes HIF-2α expression stability [9,11]. We decided to assess HIF-1α and HIF-2α expression in our glioma cells following Int6 silencing. Surprisingly, we observed decreased HIF-1α and HIF-2α expression. This was correlated with a down-regulation of some HIF target genes (*VEGF*, *PDGF-β*, *AlphaV integrin* and *Dll4*) involved in proliferation, migration and cell growth processes. These results indicate that Int6-dependent HIF-2α regulation is cell type specific and occurs in a different manner in glioblastoma cells. In human brain glioma cells, Int6 inhibition leads to decreased HIF-1α and HIF-2α expression. Despite a significant and reproducible decrease of HIF expression in the four tested glioma cell lines, HIF down-regulation following *Int6* silencing occurs in different proportions depending on the cell lines. These differences could be due to the cellular genetic/metabolic backgrounds, which lead to various responses within each cell line when Int6 is inhibited. The extent to which the changes we observed in HIF and HIF target expression are a direct or indirect result of regulation by Int6/eIF3e is an interesting question. It has been reported that Int6 specifically regulates some mRNAs. Int6 suppression inhibits translation of mRNA encoding *GADD45* or *MAP3K14* in HeLa cells [35], affecting cell growth, and stimulates translation of mRNA encoding Snail1 or Zeb2 in MCF10A cells [19], inducing epithelial-mesenchymal transition. Decreased HIFs expression in GBM cells, may be due to a diminished *HIF* mRNA translation. These decreases in HIF expression could potentially explain the phenotypes we observe after *Int6* silencing. Indeed, it is now clearly established in glioblastoma cells that HIF target inhibition induces decreased cell proliferation, migration or invasion. For instance, the Notch/Dll4 pathway inhibition has been shown to reduce the migratory and invasive properties of U87 and U251 glioma cells [36]. VEGF inhibition suppresses GBM stem-like cells tumorigenesis and angiogenesis through VEGF receptor 2 and decreases U251 and LN18 motility [37,38]. Glioblastoma cells express PDGF-β and its receptor, which both contribute to proliferation and angiogenic processes [39]. AlphaV integrin depletion in glioma cell lines impairs cell growth and invasion through induction of apoptosis [40,41]. Despite diminished HIF and HIF target expression, other mechanisms leading to decreased proliferation are likely involved in response to Int6 inhibition. However, to thoroughly characterize the degree to which the HIFs are directly involved in the defect of glioma cell proliferation after Int6 depletion, it will be essential to perform experiments under low oxygen conditions or using hypoxia mimetics, such as CoCl_2_. These investigations and the identification of new Int6/eIF3e targets are currently ongoing in our laboratory.

Finally, several studies have shown that the HIFs, particularly HIF-2α, are specifically expressed in glioma stem cells and regulate genes involved in the stemness of these cells such as *Oct4*, *c-Myc* or *Sox2* [3,8,42,43]. Interestingly, Li *et al.* showed that HIF-2α inhibition decreases neurosphere formation and induces increased glioma stem cell apoptosis [8,42]. In the present study, we showed that Int6 silencing inhibits human glioma cell proliferation via induction of cell cycle arrest and apoptosis, potentially through HIF downregulation, supporting the idea that Int6/eIF3e could favor tumor growth as described in breast cancer and osteosarcoma cells. In consequence, Int6/eIF3e modulation in glioma stem cells could be of special interest for eradication of these treatment-resistant cells and needs to be studied further.

## 3. Experimental Section

### 3.1. Cell Culture

Four human glioblastoma cell lines, U87 (ATCC HTB-14), LN18 (ATCC CRL-2610), U251 and SF767 (obtained from M. Celeste Simon’s laboratory, University of Pennsylvania, Philadelphia, PA, USA), were used and routinely maintained in DMEM supplemented with 10% fetal calf serum at 37 °C in 5% CO_2_-humidified incubators and were subcultured once or twice a week.

### 3.2. siRNA Transfection

Human glioblastoma cells (2 × 10^5^) were transfected with 20 nmol/L of siScramble (siScr, Qiagen, Venlo, Limburg, The Netherlands) or 20 nmol/L of siRNA against human *Int6/EIF3E* (siInt6, see below Table 1) combined with Lipofectamine, RNAimax reagent as recommended by the manufacturer (Invitrogen Life Technologies, Carlsbad, CA, USA).

### 3.3. Proliferation Assay

Human glioblastoma cells (2.5 × 10^4^) cells were transfected with siRNA (siScr or siInt6). Seventy-two hours after transfection, cells were collected daily for 5 days in 1 mL of trypsine. Twenty microlitres of cell suspension were mixed with 20 μL of Trypan Blue (Lonza, Bâle, Switzerland). Cells were counted using Malassez slide (Invitrogen Life Technologies, Carlsbad, CA, USA) and the number of cells per milliliter were determined by the following formula: (Number of cells/20 squares) × 2 × 100 × 1000. Pictures were taken with Nikon microscope (NIS Element, Nikon, Champigny sur Marne, France), 3 days after transfection with siRNA.

### 3.4. Western Blot Analysis

Cells, untransfected or transfected with siRNA, were lysed in 70 μL of lysis buffer (50 mM Tris HCl pH 7.5, 0.1% Triton, 5 mM EDTA complemented by Proteases and Phosphatases inhibitors (Chemicon Millipore, Billerica, MA, USA and Sigma Aldrich, Saint-Louis, MO, USA) at 1/100). Western blots were performed as previously described [44,45] using monoclonal rabbit antibodies, anti-Int6 (1/1000, Abcam, Paris, France), anti-HIF-2α (1/1000, Novus Biologicals, Cambridge, UK), anti-HIF-1α (1/1000, Cayman Chemical, Ann Harbor, MI, USA), anti-Caspase 3/7 (1/1000, Cell Signaling, Danvers, MA, USA), anti-cleaved caspase 3/7 (1/1000, Cell Signaling, Danvers, MA, USA) anti-PARP (1/1000, Cell Signaling, Danvers, MA, USA), anti-Bax (1/1000, Cell Signaling, Danvers, MA, USA), anti-Bcl-XL (1/1000, Cell Signaling, Danvers, MA, USA), anti-Bcl-2 (1/1000, Cell Signaling, Danvers, MA, USA), anti-MEK1 (1/1000, Cell Signaling, Danvers, MA, USA), anti-ERK (1/1000, Cell Signaling, Danvers, MA, USA), anti-phospho ERK (1/1000, Cell Signaling, Danvers, MA, USA), and were normalized using a mouse monoclonal antibody anti-actin (1/10,000, Chemicon Millipore, Billerica, MA, USA) or a rabbit polyclonal antibody anti-β-tubulin (1/1000, Cell Signaling, Danvers, MA, USA). Gel quantification was performed using ImageJ (Windows 1.47, Research Services Branch, NIH, Bethesda, MD, USA).

### 3.5. RT-qPCR

Total RNA was extracted using RNeasy Mini Kit (Qiagen, Venlo, Limburg, The Netherlands), treated with DNase I and subjected to reverse transcription (iScript™ cDNA Synthesis Kit, BioRad, Hercules, CA, USA). qPCR were performed with different primers (see below, Table 2) and normalized using *β2-microglobuline* and *18S* genes, using a StepOnePlus (Applied Biosystems Life Technologies, Carlsbad, CA, USA).

### 3.6. Flow Cytometry

Seventy-two hours after transfection with siRNA (siScr or siInt6) and treatment or not with 30 μM of Z-VAD-FMK caspase inhibitor (Sigma Aldrich, Saint-Louis, MO, USA), cells were collected, washed in PBS and incubated in 100 μL of Annexin-binding buffer 5× (10 mM HEPES pH7.4, 140 mM NaCl, 2,5 mM CaCl_2_), containing 5 μL of Annexin V-FITC antibody and 1 μL of Propidium Iodide (PI, Invitrogen Life Technologies, Carlsbad, CA, USA) solution at 100 μg/mL (FITC Annexin V/Dead Cell Apoptosis Kit with FITC annexin V and PI, Invitrogen Life Technologies, Carlsbad, CA, USA) during 15 min at room temperature (RT) in the dark. Four hundred microliters of Annexin-binding buffer 5× were then added after washes with PBS-BSA 1%. For cell cycle analysis, cells were fixed with cold ethanol 100% and then permeabilized with Triton ×100 at 0.25%. Cells were then labeled with Ki67 (Abcam, Paris, France) during 45 min at RT and treated with RNAse 1 μg/mL before labeling with PI during 2 hrs at RT. Labeled cells were preserved on ice and run on a flow cytometer (FACS Calibur, Becton-Dickinson, Franklin Lakes, NJ, USA).

### 3.7. *De Novo* Protein Synthesis

Cells were starved with methionine/cysteine free medium +10% dialyzed serum for 1 h and then, incubated with ^35^S methionine (80 μCi/mL) for 30 min in order to assess *de novo* protein synthesis. Proteins were resolved by SDS-polyacrylamide gel, either dried and exposed to Biomax films (Kodak, Carestream Health, Rochester, NY, USA) or stained with Coomassie (Biorad, Hercules, CA, USA). The *de novo* synthesis of protein was detected by autoradiography.

### 3.8. Migration/*in Vitro* Wound-Closure Assay

For the wound-closure assay, human glioma cells transfected with siScr or siInt6 were seeded in 6 cm dishes and incubated overnight to generate confluent cultures. Of note, U87 cells do not form a monolayer of cells and we were therefore unable to perform the test for these cells. Cell layers were scraped with a plastic pipette tip and washed three times with serum-free media. The remaining cell culture was incubated 24 h to allow cells to migrate into the cleared space. To quantify cell migration, phase-contrast images of identical locations in each wound were taken at 0, 13 and 20 h after wounding. The rate of cell migration was then calculated as the average percentage of wound closure from at least three independent experiments using Nikon microscope and Image J software (Windows 1.47, Research Services Branch, NIH, Bethesda, MD, USA).

### 3.9. Statistical Analysis

Student’s test was done to compare the means of values from different experiments. Differences were considered statistically significant at *p* < 0.05. (* p < 0.05, ** p < 0.01, *** p < 0.001).

## 4. Conclusions

In conclusion, our study demonstrated that Int6, also known as eIF3e, plays an essential role in human glioblastoma cells controlling cell growth, cell cycle, and cell death. We highlighted that Int6/eIF3e inhibition through siRNA induces a diminution of glioblastoma cell proliferation through cell cycle arrest and an induction of caspase-dependent and caspase-independent cell death (Figure 7). With no modification in global cell translation following Int6 inhibition, the observed phenotypes could be, in part, explained by the decrease of HIF and HIF target expression in GBM cells. Although a deeper understanding of the molecular mechanisms involved in this Int6/eIF3e-HIFs pathway is necessary, Int6 could become a new therapeutic option for these aggressive tumors.

## Supplementary Information



## Figures and Tables

**Table 1.** *Int6/EIF3E* siRNA sequences.

Source	Sequence
Qiagen FlexiTube Gene Solution GS3646 for *EIF3E*, 1 nmol	SI02662499: 5′-CCCAAAGGUCGCGAUAAUAUU-3′ SI02661981: 5′-AAGCUGGCCUCUGAAAUCUUA-3′ SI02656094: 5′-AAGCUGAAAGGUGGAUUGUAA-3′ SI03153136: 5′-AUGGAAGACCUUACACGGUUA-3′
Qiagen SI02662499 (FlexiTube *EIF3E* siRNA, 20 nmol)	5′-CCCAAAGGUCGCGAUAAUAUU-3′
Dharmacon (ON-TARGET plus SMART pool *EIF3E* siRNA 3646, 10 nmol)	#1: 5′-UGGCUUGUCUUGAGGAUUU-3′ #2: 5′-GGAUCGGCAUCUAGUCUUU-3′ #3: 5′-GGGUAACAAUGCAGUCUCA-3′ #4: 5′-AAAGGUCGCGAUAAUAUUA-3′

**Table 2.** qPCR primer sequences.

Targets	Forward primer (5′-3′)	Reverse primer (5′-3′)
β2m	ACCCCCACTGAAAAAGATGA	ATCTTCAAACCTCCATGATG
18S	GTCTGTGATGCCCTTAGATG	CGTACAGGATGATGTCCGTATACCT
Int6	TTCTTCAATCACCCCAAAGG	TAGAACCTGCCGACGTTTTC
HIF-2α	CCACCAGCTTCACTCTCTCC	TCAGAAAAAGGCCACTGCTT
Dll4	TGGGTCAGAACTGGTTATTGGA	GTCATTGCGCTTCTTGCACAG
PDGFβ	GATCCCTCCTTTGATGATCTC	TCCAACTCGGCCCCATCT
VEGF	CTACCTCCACCATGCCAAGT	GCAGTAGCTGCGCTGATAGA
αV Integrin	GGAGCAATTCGACGAGCACT	TTCATCCCGCAGATACGCTA
