# Supplementary material for: Int6/eIF3e Is Essential for Proliferation and Survival of Human Glioblastoma Cells
Source: Int J Mol Sci. 2014 Jan 29;15(2):2172–90. doi: 10.3390/ijms15022172 (PMC3958844; doi:10.3390/ijms15022172)

Supplementary Information

Figure S1. *Int6* gene silencing efficiency. (**A**) Western Blot analysis of Int6 expression at different times after siRNA transfection. Int6 expression is strongly silenced in *Int6* knockdown GBM cells (siInt6) compared to negative control (siScr) at 24 h, 48 h, 96 h and 7 days after siRNA transfection; (**B**) Western Blot analysis of Int6 expression using different siRNA concentrations. Int6 expression is inhibited with siInt6 treatment starting at 1 nM, 5 nM and 50 nM of siRNA in all glioma cell lines; (**C**) Western Blot analysis of HIF-1α and HIF-2α expression after Int6 inhibition. HIF-1α and HIF-2α expression is reduced in human GBM cells where Int6/eIF3e is inhibited (siInt6); (**D**) Western Blot analysis of Int6 and HIF-2α expression after transfection with other siRNA sequences against *Int6* (siInt6, Dharmacon On-Target plus Smart pool). Int6 inhibition is confirmed as well as the decreased HIF-2α expression in human GBM cells when *Int6/EIF3E* is silenced; (**E**) Western Blot analysis of Int6, HIF-1α and HIF-2α expression after transfection with siInt6 from Qiagen Pool FlexiTube Gene Solution (Qiagen, Venlo, Limburg, Netherlands). Int6 inhibition is confirmed as well as the decreased HIF expression in human GBM cells when *Int6/EIF3E* is silenced.

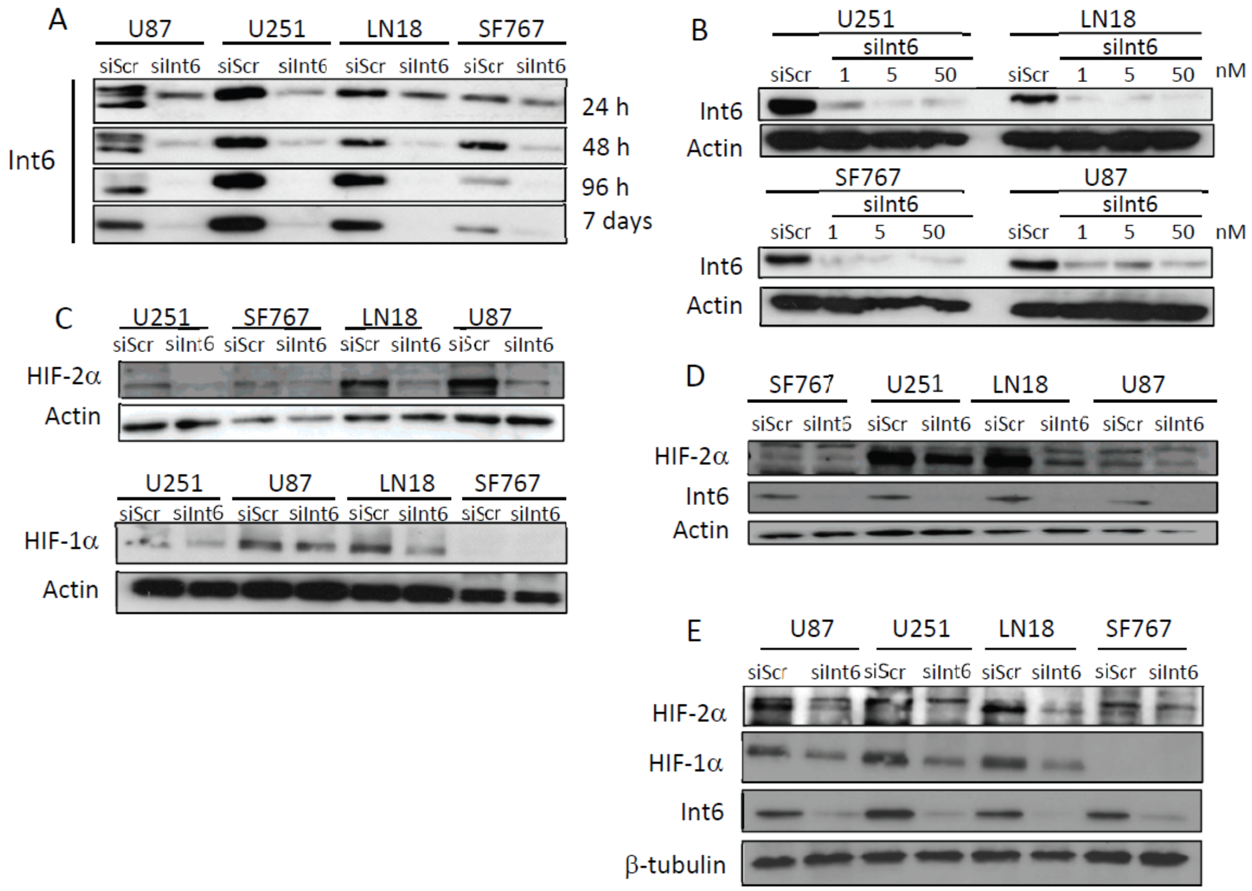

Figure S2. Int6 inhibition with different and distinct siRNA sequences also alters human glioma cell proliferation. Proliferation assay with GBM cells transfected or not with (**A**) *Int6* siRNA (siInt6, Dharmacon On-Target plus Smart pool) or (**B**) *Int6* siRNA (siInt6, Qiagen Pool FlexiTube Gene Solution, Qiagen, Venlo, Limburg, Netherlands) shows a significant decreased GBM cell growth when Int6 is inhibited compared to the negative control group (* $p < 0.05$, ** $p < 0.01$, *** $p < 0.001$ siInt6 versus siScr, $n = 3$).

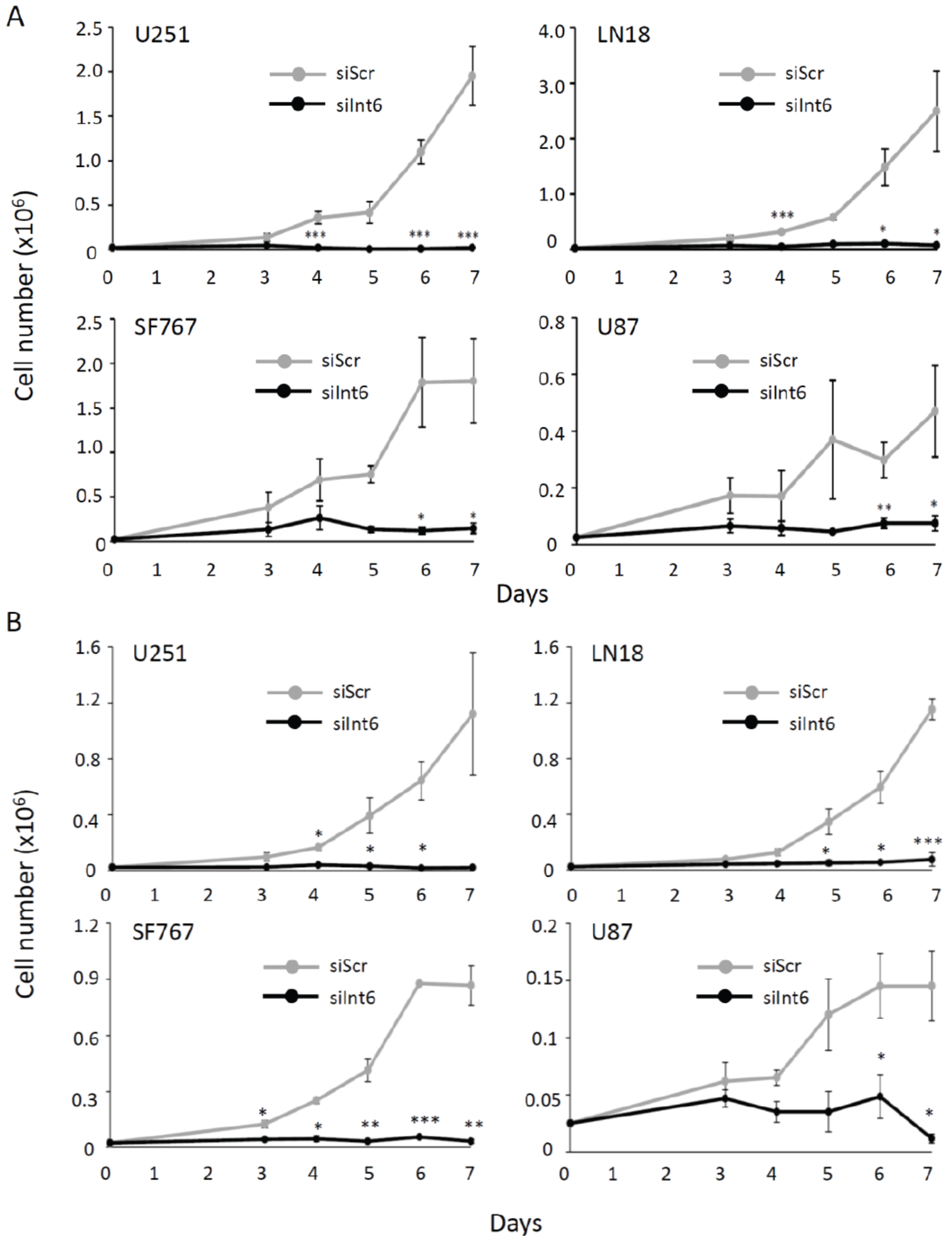

Figure S3. *Int6* gene silencing haltes cell cycle in glioma cells. (**A**) Representative dot plots of cell cycle distribution in four GBM cell lines (U87, U251, LN18 and SF767). FL2-H represents the Propidium Iodide staining; (**B**) Representative dot plots of GBM cells in G0 phase. *Int6* knockdown (siInt6) significantly increases the number of Ki67 negative cells compared to negative control group (siScr), n = 4. FL1-H represents Ki67 staining and FL2-H represents Propidium Iodide staining.

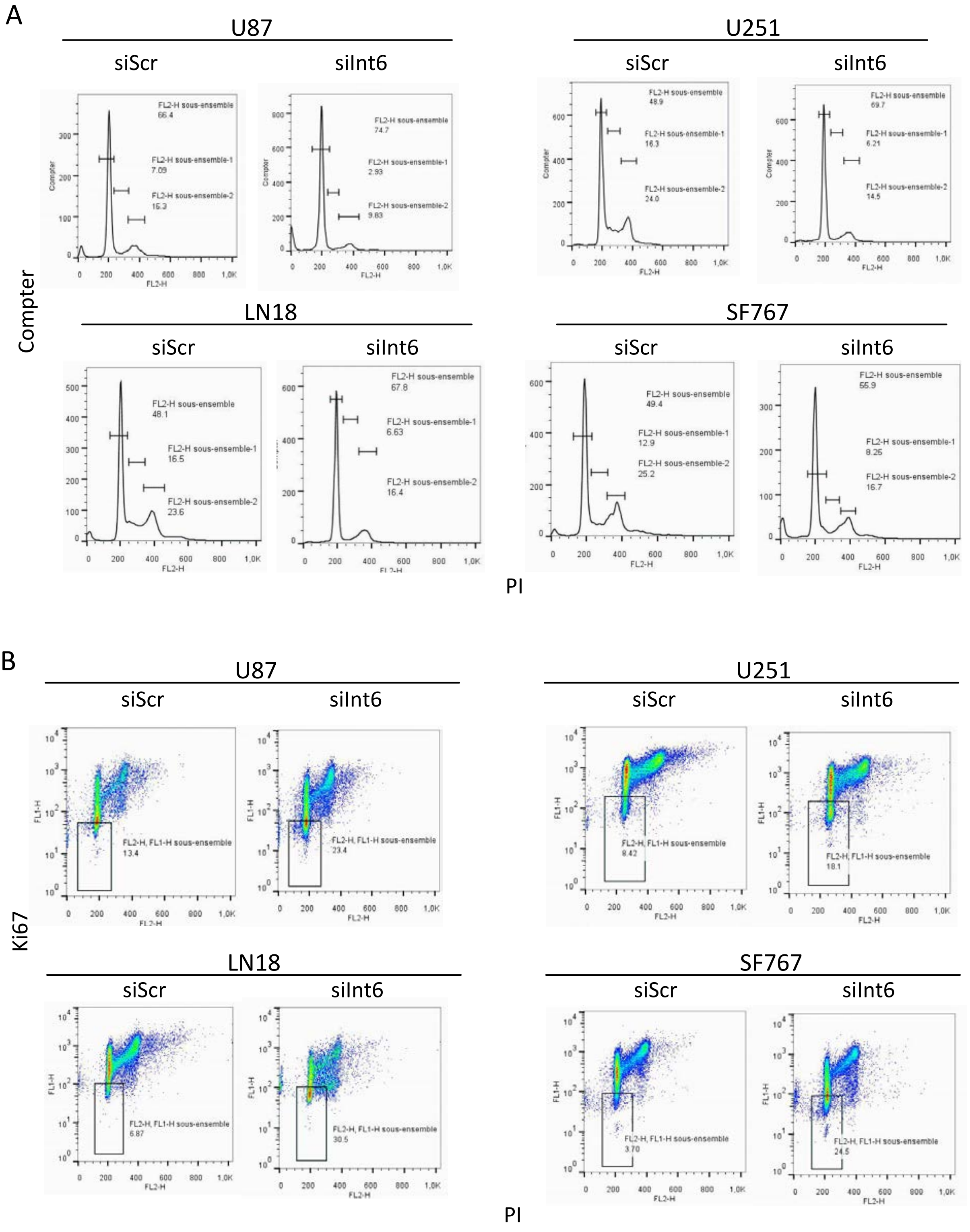

Figure S4. *Int6* gene silencing induces human glioma cell apoptosis. (**A**) Representative dot plots showing percentage of Annexin V positive cells in GBM cell lines (U251 and SF767). FL1-H represents Annexin V-FITC staining and FL2-H represents Propidium Iodide staining; (**B**) Western Blot analysis of caspase 3 and PARP expression in GBM cells following *Int6* silencing. Caspase 3 and PARP expressions are reduced in *Int6* knockdown group compared to negative control group (siScr); (**C**) Western Blot analysis of, the pro-apoptotic protein, Bax expression in GBM cells. Bax expression increases in all glioma cell lines after transfection with different siInt6 concentrations (1, 5 or 50 nM) compared to negative control group (siScr).

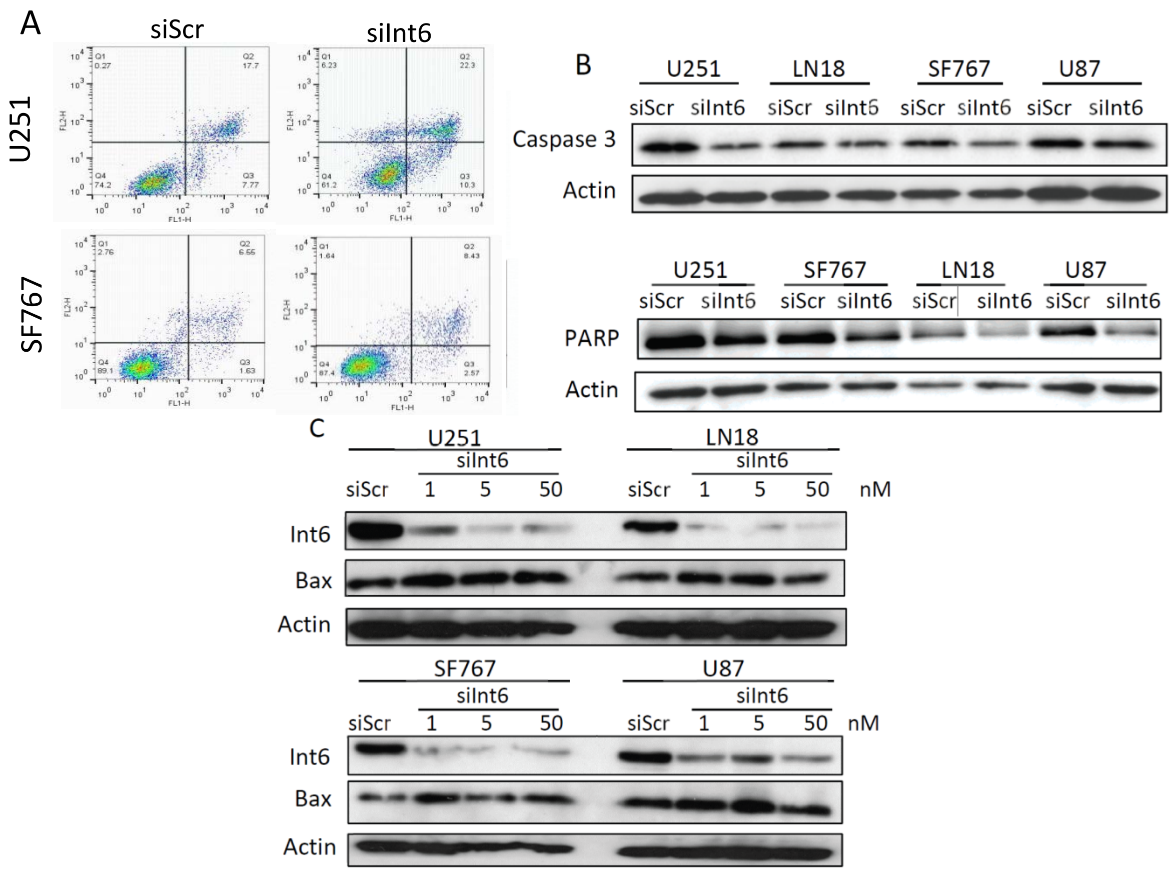

Figure S5. *Int6* gene silencing does not alter global GBM cell translation. (**A**) Total proteins of the same number of glioma cells transfected or not with siInt6 (72 h after transfection) were resolved by SDS-polyacrylamide gel and stained with blue Coomassie. Lane M, molecular weight marker; at the bottom of the gel is indicated the number of cells loaded for each cell line. *De novo* protein synthesis in U251 cells was assessed, 24 h (D1), 48 h (D2) and 72 h (D3) after transfection with siInt6 or control siRNA (siScr) using ^{35}S metabolic labeling (**B**) and Coomassie blue staining (**C**), *n* = 3.

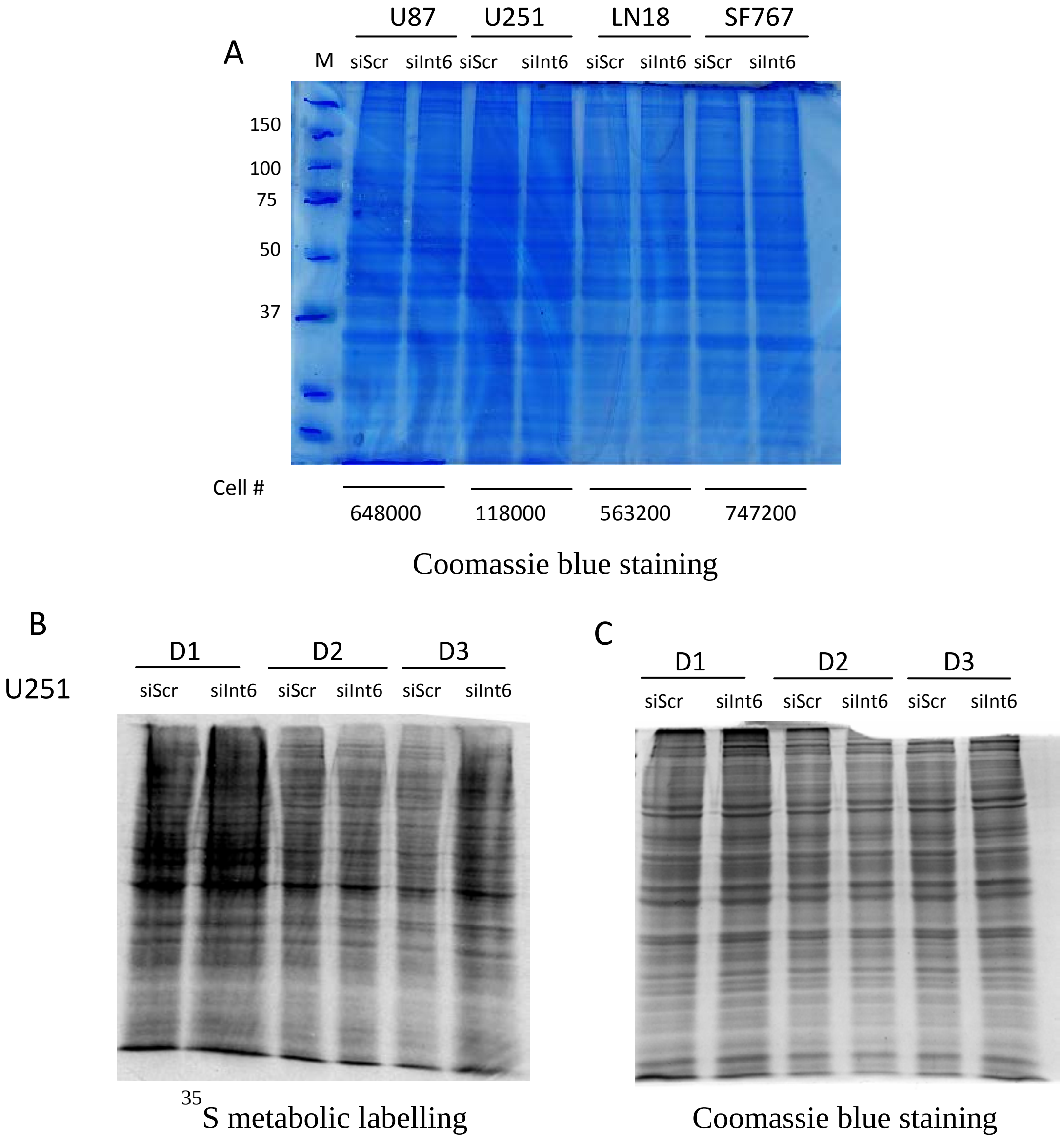

Figure S6. Int6 deletion affects human glioma cell migration. (**A**) Migration of control cells (siScr) or cells where Int6 is inhibited (siInt6) was assessed using a scratch wound assay. Test was performed with LN18, SF767 and U251 cell lines. Of note, U87 cells do not form a monolayer of cells and we were therefore unable to perform the test for these cells. Representative photographs shown in (**A**) were taken after the scratch (T0), and 20 h later (T20). We selected time points <24 h to avoid the effect of Int6 inhibition on proliferation (**B**) Percentage of wound closure was determined, 13 h (T13) and 20 h (T20) after wound, at consistent locations and was significantly decreased for siInt6 cells compared to siScr control cells at 20 h (* $p < 0.05$, ** $p < 0.01$, *** $p < 0.001$, $n = 3$).

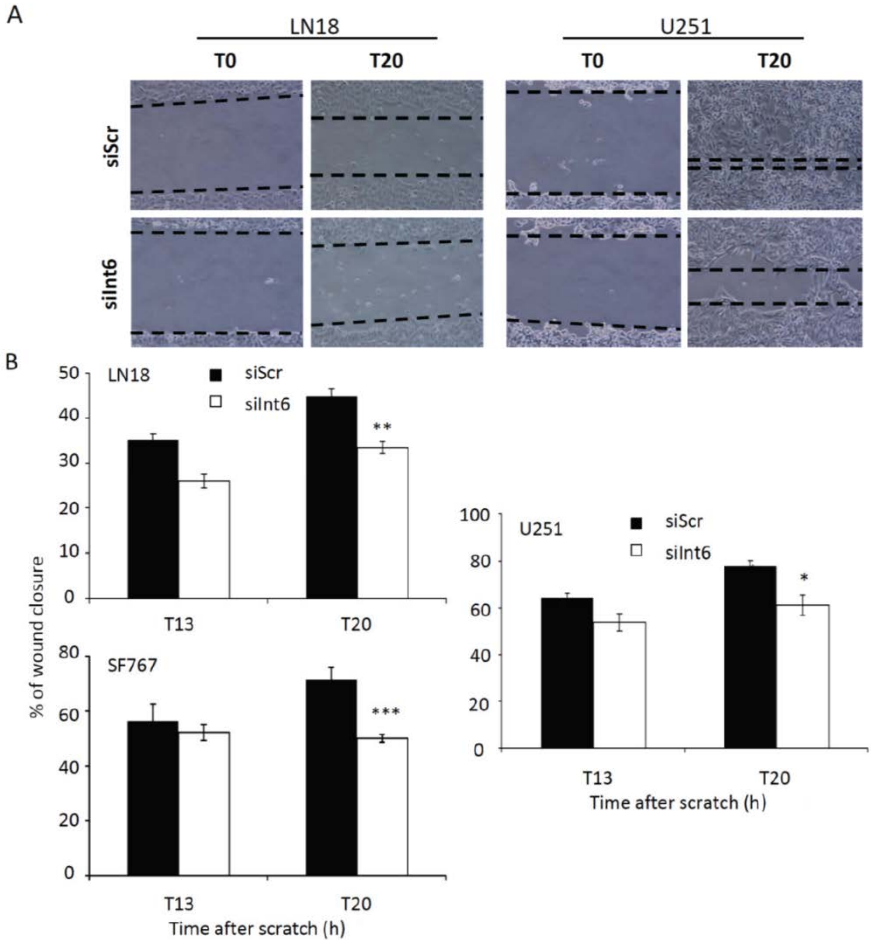

Figure S7. *Int6* gene silencing does not alter MAPK pathway in GBM cells. Western Blot analysis of MAPK pathway (MEK1, ERK and phospho-ERK) in glioma cells after Int6 depletion, $n = 3$.

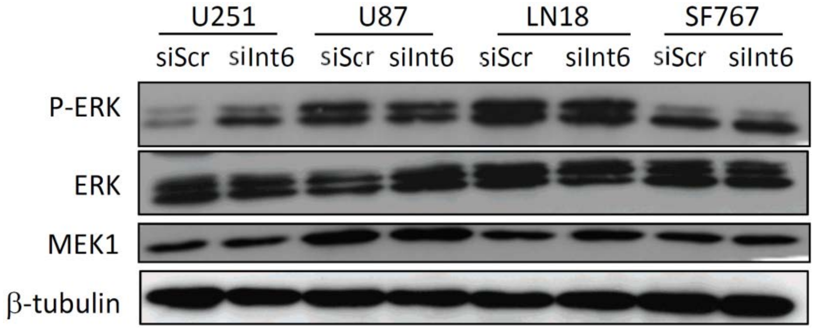

Supplement: Supplementary file 1 [file ijms-15-02172-s001.pdf]
